# Endogenesis and Externalization: Configurational Influence of Learning Engagement Among Chinese University Students Majoring in Tourism

**DOI:** 10.3390/bs15020111

**Published:** 2025-01-22

**Authors:** Fenglong Yu, Qian Chen, Bing Hou

**Affiliations:** School of Tourism and Cuisine, Yangzhou University, Yangzhou 225127, China; qian.chen@yzu.edu.cn (Q.C.); bhou@yzu.edu.cn (B.H.)

**Keywords:** learning engagement, configurational influence, internal and external factors, university students majoring in tourism, China

## Abstract

Learning engagement among university students is a critical predictor of academic success. This study, drawing on responses from 333 questionnaires completed by Chinese tourism students, employs the fsQCA method to construct a configurational impact model of learning engagement, exploring the paths and mechanisms of its influence. The study finds that learning engagement among tourism students is shaped by the combined influence of internal and external factors, with internal factors—such as professional cognition, professional evaluations, professional emotions, and academic self-efficacy—playing a foundational and central role. External factors, such as the university environment, provide additional influence, though their impact varies depending on the type of learning engagement. A high level of learning engagement is associated with two distinct configurational paths, identified as the endogenous model and the endogenous–exogenous promotion model. Having positive professional evaluations and a strong professional identity is found to have a significant positive impact on students’ academic engagement. Conversely, a low level of learning engagement follows three distinct configurational paths, collectively termed the endogenous suppression model, in which a lack of professional emotions and low academic self-efficacy are key inhibitors of academic engagement. Theoretical and practical implications based on the research findings are also discussed.

## 1. Introduction

The high-quality development of tourism higher education in China is currently a critical task for advancing tourism education (Bao, 2023). China’s national leader, General Secretary Xi Jinping, has emphasized that the rapid growth of China’s tourism economy has paved a unique path for development. The progress of tourism education in China has mirrored this economic expansion, growing to become the world’s largest tourism education system, and establishing a distinctive approach to cultivating tourism talent with Chinese characteristics. However, the compounded effects of the downturn in the tourism economy and the COVID-19 pandemic have presented significant challenges. Many undergraduate universities in China are now facing issues such as the shrinking of tourism programs, low employment rates within the industry, and the loss of talent from tourism enterprises. Consequently, undergraduate students majoring in tourism are increasingly doubtful about their professional development prospects, exhibit a weak professional identity, and lack sufficient learning engagement (Bao, 2023; S. Yin et al., 2022).

Learning engagement is a direct predictor of learning performance and a critical factor influencing future development, making it a prominent topic in educational research (H. Yin, 2016). In the 1980s, the concept of ’learning engagement’ was introduced for the exploration of effective teaching and learning quality (Astin, 1984), and it began attracting the attention of Chinese scholars by the end of the 20th century (Guo et al., 2021). Zepke (2014) noted that learning engagement appears to be a universally accepted and unquestioned academic belief. The issue of insufficient student engagement among Chinese university students has garnered widespread attention and has become a key factor limiting the transformation of Chinese higher education from ’large’ to ’strong’ (Y. Li & Liu, 2016; Long & Ni, 2020). Numerous studies have shown that increasing learning engagement helps reduce negative emotions towards learning and enhances students’ satisfaction with their educational experience (H. Yin, 2020).

Issues in Chinese tourism education have become a significant research focus, encompassing multiple aspects such as curriculum design, program development, talent cultivation, and professional identity (Q. Chen et al., 2024; W. Wang & Wang, 2021). Research indicates that Chinese university students majoring in tourism generally exhibit low levels of engagement and often adopt passive learning habits, including difficulties with self-directed learning (Bao, 2023; Yu et al., 2021). In 2019, China’s Ministry of Education issued the “Implementation Opinions on the Construction of First-Class Undergraduate Courses”, which clearly stated the need to “increase learning engagement”, formally incorporating this educational concept into national policy discourse. As Jones (2003) stated, higher education must give adequate attention to students’ learning performance, as ensuring the learning quality of university students is a core objective of higher education institutions. However, compared to the breadth and depth of learning engagement research in other disciplines, relatively little academic attention has been paid to the learning engagement of Chinese undergraduate students majoring in tourism. Research on learning engagement is destined to become a focal point and significant area of interest in the field of tourism higher education in China.

This study explores the internal and external factors influencing learning engagement among Chinese undergraduates majoring in tourism from the perspective of configurational theory. Its specific contributions are reflected as follows. Firstly, the research uses the fsQCA method to develop a configurational impact model of learning engagement among tourism undergraduates, considering both internal factors (professional cognition, professional evaluations, professional emotions, and academic self-efficacy) and external factors (the university environment). Secondly, the level of learning engagement is likely the result of the combined influence of internal and external factors, but the degree of impact from different factors may vary. Thirdly, this study aims to explore the configurational impact pathways of high and low levels of learning engagement among undergraduates majoring in tourism, as well as the role of internal and external factors in different paths. To achieve the research objectives and goals, the remainder of the paper is structured as follows. First, based on a literature review, the paper analyzes the concept of learning engagement and its relationships with factors such as professional identity, academic self-efficacy, and the university environment. Second, the paper outlines the research design. Based on an analysis of the research methods, it constructs an analytical framework for the configurational impact on learning engagement. It also introduces the measurement tools and data sources. Third, using the fsQCA method, the paper explores the factors influencing learning engagement, summarizing and elaborating on the configurational impact paths. Finally, the results are analyzed, theoretical contributions and practical implications are discussed, and the study’s limitations and future research directions are presented.

## 2. Theoretical Foundation and Analysis Framework

### 2.1. Learning Engagement

Kuh (2001) proposed that the extent of learning engagement is the core element of educational quality. He succinctly defines learning engagement as “the time and energy that college students invest in activities with educational purposes”. The connotations and dimensions of learning engagement form the foundation of this research and are also a prominent topic (Guo et al., 2021). Learning engagement is intrinsically linked to nearly all aspects of student learning, contributing to its complexity and multidimensionality. Kahu (2013) categorized existing research on learning engagement into four perspectives: behavioral, psychological, sociocultural, and holistic. The behavioral perspective emphasizes the time and effort that undergraduate students spend in activities with educational purposes. The psychological perspective views learning engagement as a multidimensional psychological structure that includes behavioral, emotional, and cognitive aspects. The sociocultural perspective focuses on the influence of broader sociocultural contexts in defining and measuring learning engagement. The holistic perspective attempts to integrate these viewpoints.

Students’ overt engagement behaviors and implicit motivational states are inseparably linked. Martin (2009) proposed a conceptual framework called the “Motivation and Engagement Wheel” (MEW), viewing learning engagement as a complex, multidimensional structure, and this framework aligns with the psychological standard that learning engagement should encompass behavioral, emotional, and cognitive aspects, which are defined as follows. Behavioral engagement refers to the extent of students’ participation in academic, social, and extracurricular activities. Emotional engagement concerns the emotional identification and degree of connection students have with their teachers, peers, courses, and the institution. Cognitive engagement reflects the intellectual investment and willingness of students to master complex concepts or skills (Fredricks et al., 2004; Parsons et al., 2014). Sociocultural engagement has a significant impact on learning engagement (Kahn, 2014); however, the factors and mechanisms involved in the sociocultural dimension are complex. Therefore, this study focuses on exploring the factors influencing learning engagement from the perspective of intrinsic psychological factors and external behavioral factors. Skinner et al. (2008) proposed a self-system model of motivational development (SSMMD) to explain the internal and external mechanisms influencing learning engagement. This model consists of an internal system and an external system. The internal system includes emotional and behavioral engagement. Positive emotions (such as interest and pleasure) promote positive behaviors (such as effort and persistence), while negative emotions lead to negative behaviors (Manwaring et al., 2017; Sun & Rueda, 2012).

Research shows that the factors influencing learning engagement among Chinese university students differ from those in Western countries (H. Yin, 2020). It involves not only balancing internal psychological states and external engagement behaviors but also considering the sociocultural context. Chinese undergraduate students display a “discrepancy between internal and external” factors in their learning motivation and engagement, which may differ significantly from the experiences of undergraduates in Western countries. This indicates that understanding undergraduates’ learning engagement requires consideration of both their internal psychological states and external behavioral involvement. Additionally, it is crucial to acknowledge the sociocultural context in which students’ learning takes place. In China, this context is profoundly shaped by cultural and educational traditions that prioritize diligence and perseverance, as well as by unique cultural constructs such as “shame” and “guilt” (Watkins, 2000). Specifically, the factors influencing learning engagement can be categorized into two aspects: the external environment and the internal environment (W. Wang & Wang, 2021). A supportive university environment helps enhance students’ self-perception, which promotes learning engagement and ultimately improves learning outcomes.

With the advancement of internet technology, increasing research has focused on assessing students’ psychological and behavioral engagement, emphasizing the crucial roles of both psychological and behavioral involvement (L. Q. Su, 2020). Addressing the prominent issues of insufficient initiatives for learning engagement among Chinese students and the inconsistency between their psychological and behavioral aspects (H. Yin, 2020), this article examines students’ internal psychology, mental engagement in learning, and external behavioral engagement. It emphasizes students’ subjective initiative, vigor (perseverance during the learning process), dedication (strong enthusiasm for learning and self-esteem), and focus (concentration on learning and deriving pleasure from it) as measurement scales. Additionally, influenced by and building on research findings such as Skinner’s SSMMD model (Skinner et al., 2008), this paper explores the relationships between university students’ professional identity (including their professional cognition, professional evaluations, and professional emotions), academic self-efficacy, the university environment, and learning engagement, considering both internal and external factors.

### 2.2. Professional Identity and Learning Engagement

Professional identity focuses on the internal psychological factors influencing learning engagement. Social identity theory emphasizes an individual’s sense of belonging to a social category or group and how this sense of belonging influences individual behavior (Gomez & Vazquez, 2015). Based on similarities and differences within and between groups, individuals identify with or distinguish themselves from their group in terms of cognition, emotion, and behavior (Hogg, 2000). Therefore, social identity theory helps to understand university students’ professional identity through the lenses of cognition, evaluation, and emotion (Smilde, 2016). Specifically, professional cognition refers to the process by which students understand their field of study through acquiring professional knowledge. Professional evaluations refer to positive or negative evaluations of the fit between the individual and the group, while professional emotions refer to personal attachments to the group and its members (Ashforth & Mael, 1989). The dimension of professional emotions refers to an individual’s attachment to the tourism major, which is manifested in professional confidence, employment intention, emotional belonging, and satisfaction degree. Social identity theory emphasizes that social emotions are stimulated through self-cognition, evaluation, and categorization, which is not only a dynamic process but also a relatively stable outcome. This bears certain similarities to the connotations and structure of learning engagement (Skinner et al., 2008) and provides theoretical insights for exploring the relationship between professional identity and learning engagement from a psychological perspective.

Professional identity is a key indicator of learners’ personal values and career development goals (Richardsonsupa, 2010). Research indicates that professional identity plays a crucial role in breakthroughs within the tourism discipline and in the high-quality development of tourism education (Bai et al., 2012), which directly affect students’ learning performance and future career choices (J. Wang, 2018; C. H. Wang et al., 2020). Specifically, university students majoring in tourism develop professional cognition by mastering tourism theories and industry knowledge. Based on their understanding of the tourism profession, these students assess their alignment with the profession and form positive or negative evaluations (Yu et al., 2021). This process leads to emotional acceptance and attachment to the tourism profession, which in turn promotes their learning engagement (Parker et al., 2021). Conversely, it may also result in the opposite effect. The understanding of professional identity described above can be summarized as a dynamic and gradual process composed of three dimensions: professional cognition, professional evaluations, and professional emotions. Building on this, this paper explores the impact of professional identity on learning engagement, focusing on analyzing the differentiated effects and interactions of the various dimensions (cognition, evaluations, and emotions) on learning engagement.

### 2.3. Academic Self-Efficacy and Learning Engagement

Academic self-efficacy refers to an individual’s judgment and evaluation of their ability to perform specific tasks. This concept originates from the theory of self-efficacy introduced by Bandura (1989). Bandura (1989) defined self-efficacy as “the degree of confidence people have in their ability to use their skills to complete a task”. If individuals believe that a specific behavior will lead to a certain outcome, this behavior is more likely to be initiated and chosen (Bandura, 1989). The higher one’s self-efficacy—meaning the greater an individual’s belief in their ability to succeed in a particular area—the more they will strive to actively engage in a task (Lin et al., 2020).

Academic self-efficacy extends and concretizes the theory of self-efficacy within the context of learning activities, specifically referring to students’ judgments and evaluations of their ability to complete specific academic tasks and achieve particular academic goals (W. Li & Bai, 2018). To some extent, academic self-efficacy reflects university students’ self-regulated learning (SRL) abilities (Guo et al., 2021). Grounded in theories such as information processing, social cognition, and sociocultural perspectives, SRL encompasses dimensions such as cognition, motivation, behavior, and emotion. Researchers in SRL posit that learning is a self-constructed process in which students actively monitor, control, regulate, and manage their own thoughts, feelings, and behaviors to achieve self-set learning goals. Students with stronger self-regulated learning abilities are more likely to achieve academic success (Mínguez et al., 2021). Additionally, this learning process is influenced by both individual and environmental factors (Richardson, 2007).

Ferla et al. (2010) studied the impact of self-concepts such as academic self-efficacy on learning styles and academic performance, demonstrating that student efficacy can affect students’ academic engagement and performance. Academic self-efficacy is an individual’s evaluation of their own abilities, which in turn influences their choices and specific behaviors. This has been supported by numerous empirical studies, which indicate that students with higher academic self-efficacy often achieve better academic results, even when their innate qualities and external environments are similar (Q. Chen et al., 2024; W. Li & Bai, 2018). In summary, students with higher academic self-efficacy tend to employ more in-depth cognitive and metacognitive strategies, resulting in better learning outcomes.

### 2.4. University Environment and Learning Engagement

The university environment encompasses external aspects such as the support provided by the university in terms of courses, teachers, degrees, and employment, as well as the learning atmosphere it fosters. This study defines “environment” as the learning support and atmosphere perceived by students at their university. Students’ subjective perception of the learning environment has a more direct impact on learning than the objective environment itself (Guo et al., 2021). The university environment is crucial for students’ academic and social-emotional development. Students can transform external favorable or even unfavorable factors influencing learning into internal learning motivation, depending on their perception of the environment (Q. Chen et al., 2024). Kuh (2009) proposed that learning engagement includes not only the time and effort students invest in academic and effective educational activities but also how they perceive the support provided by the university for their learning (Gao et al., 2020). Therefore, learning engagement is influenced not only by individual factors but also by external factors such as the university environment and atmosphere (Azila-Gbettor et al., 2023; Tian, 2018). Students who perceive higher levels of support also have higher levels of academic self-efficacy.

Maslow’s (1943) hierarchy of needs theory suggests that the need for belonging and love is a crucial psychological requirement and a prerequisite for self-fulfillment. The more a school creates conditions that encourage and support students’ active participation in effective learning activities, the more time and effort students will invest, leading to better learning outcomes (Zhang & Li, 2018). Some scholars refer to this impact as a sense of school belonging, meaning that students identify with their school and experience feelings of warmth and pride towards it (W. Li & Shi, 2021). This sense of belonging is a specific indicator of the psychological connection between university students and their school. Having a sense of belonging at their university not only affects their learning motivation but also influences their level of learning engagement (Gillen O’neel, 2021; Zhang & Li, 2018).

Since 2000, the National Survey of Student Engagement (NSSE), initiated by Kuh (2001), has been conducted annually. A research team developed a Chinese version of the survey, known as NSSE-China (Luo et al., 2009). Both the NSSE and NSSE-China surveys examine not only what students do—i.e., the time and effort they invest in activities—but also what universities do—i.e., whether and how universities implement effective measures to engage students in various activities. Therefore, it is crucial to acknowledge the support that universities provide for students’ learning engagement.

## 3. Methodology

### 3.1. Study Method

This study employs the fsQCA method, which is suitable for analyzing multiple concurrent factors and their complex relationships (Ragin, 2008). Based on an analysis of the problem context, the fsQCA adopts a holistic perspective, treating each case as a “configuration” of condition variables. It explores the causal relationships between condition configurations and outcomes through case comparison. This approach can be used to explore causal mechanisms and construct theoretical frameworks in the field of education (X. Chen & Yang, 2020), providing theoretical and methodological support for the research questions in this study.

**Figure 1:**
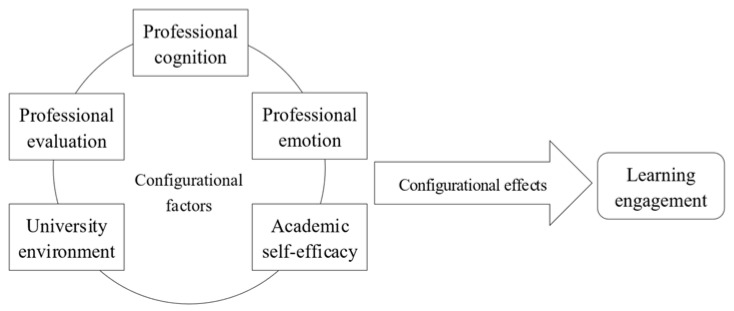
Analysis framework of configurational influence of learning engagement among university students majoring in tourism.

This study employs fsQCA to explore the configurational effects on learning engagement. The applicability of fsQCA is as follows. First, the factors influencing students’ learning engagement include various internal and external elements. The manifestation and coordination of these factors differ among students, driving variations in their learning engagement and performance through distinct paths. The configurational perspective and qualitative comparative analysis method align with the research needs, revealing the complex relationships between conditions and outcomes. Second, the variables in this study are continuous and cannot be easily categorized using binary values. The fsQCA allows for precise calibration of variables, enabling scientifically robust analysis results. Third, fsQCA addresses the explanatory challenges of quantitative analysis by highlighting various configurational conditions and explaining different configurational phenomena. This study uses five factors as antecedent conditions for fsQCA, with learning engagement as the outcome variable, constructing a configurational influence analysis framework for the learning engagement from a configurational perspective (see Figure 1).

### 3.2. Survey Instrument

(1) Professional Identity Scale: The scale for measuring students’ professional identity is adapted from various research sources (Dick et al., 2004; Yu et al., 2021). In the context of tourism higher education in China, this scale includes three dimensions with 19 items: professional cognition, professional evaluations, and professional emotions. Tourism undergraduate students’ professional identity is understood as a psychological process that includes professional cognition, professional evaluations, and professional emotions. Based on their understanding of the tourism major, students assess their fit with the profession and develop positive or negative evaluations, representing a progressive dynamic process from psychological states to behavioral intentions. (2) Academic Self-Efficacy Scale: Academic self-efficacy is assessed through 10 items adapted from the validated Chinese version of the General Academic Self-Efficacy Scale developed by Schwarzer et al. (1997). The self-efficacy scale measures two dimensions (self-efficacy beliefs and self-efficacy behaviors). Self-efficacy beliefs involve a strong conviction in one’s ability to find solutions and overcome problems. Self-efficacy behaviors specifically include the belief that one will take effective actions to successfully achieve goals when facing difficulties. (3) University Environment Scale: This scale measures the university environment from the students’ perspective and is adapted from a scale developed by Saeed et al. (2015). It includes 13 items that cover aspects such as curriculum and internships, work and further education, facilities and atmosphere, and teacher–student relationships. The university environment scale focuses on measuring students’ perceptions of university support and their sense of belonging to the university. (4) Learning Engagement Scale: To measure learning engagement, a scale adapted from the Utrecht Work Engagement Scale for Students (UWES-S) is used (Schaufeli et al., 2002). Many studies have demonstrated the reliability and validity of the UWES-S scale for Chinese university students (X. Li & Huang, 2010; Lin et al., 2020; Yu et al., 2021). The scale includes three subscales: vigor, dedication, and absorption, each evaluated by 3 items. For analysis and following previous recommendations, the 9 items from the three subscales are treated as a single predictor of learning engagement. The specific measurement items are shown in Appendix A.

### 3.3. Data Collection

The sample for this study consists of university students currently enrolled in the tourism management program at Yangzhou University, a public, regional university located in Jiangsu Province, China. Yangzhou University is a comprehensive institution offering a wide range of programs, including tourism management, and is recognized as a key university in the region. The tourism management program at Yangzhou University currently enrolls approximately 450 students. The study employed a snowball sampling method. Initially, the research team contacted tourism management students at Yangzhou University, who were then asked to assist in recruiting participants by sharing the online survey link with other students studying tourism management at universities across China. This approach allowed the sample to expand geographically, including students from multiple universities. The programs included in the study are primarily tourism management at universities across China.

To ensure that only tourism management students participated, a screening question was included at the beginning of the survey to confirm that respondents were enrolled in tourism management program. This ensured the sample was aligned with the study’s focus. Additionally, to avoid duplicate responses, we used unique survey links for each participant and monitored for repeated submissions through IP address checks during data collection. Any duplicate responses were removed during the data cleaning process. While snowball sampling was effective in reaching students across a wide range of institutions, it is important to acknowledge that this method may introduce sampling bias, as it relies on referrals from initial participants, which may lead to a non-random sample. The sample may not fully represent the broader population of tourism management students across China.

The descriptive statistical analysis showed that among the 333 respondents, females accounted for 75.80%, and 24.20% were males. This finding was consistent with previous research that there are more female students than male students in Chinese tourism programs (Yu et al., 2021). In terms of grade distribution, junior and senior respondents accounted for 26.70% and 22.20%, respectively, while freshman and sophomore respondents accounted for 21.10% and 30.00%, respectively. Missing data for individual cases were replaced with the sample mean.

### 3.4. Reliability and Validity Analysis

The results showed that the Cronbach’s α coefficients for the antecedent variables ranged from 0.900 to 0.962 (all greater than 0.7). The Cronbach’s α coefficients are as follows: professional cognition (0.900), professional emotions (0.924), professional evaluations (0.921), academic self-efficacy (0.942), university environment (0.962), and learning engagement (0.910). The composite reliability (CR) values ranged from 0.902 to 0.962 (all greater than 0.7). The CR values are as follows: professional cognition (0.902), professional emotions (0.927), professional evaluations (0.922), academic self-efficacy (0.943), university environment (0.962), and learning engagement (0.914). Additionally, the KMO value for the variables was greater than 0.7, the factor loadings for all items were above 0.711, the cumulative variance contribution rate was 89.26%, and the average variance extracted (AVE) values were all above 0.5. These results indicate that the questionnaire scales have high levels of convergent validity and internal consistency.

## 4. Results

### 4.1. Variable Calibration

Given the research context and the characteristics of the questionnaire data, the scales are typically calibrated using a direct calibration method (Du & Jia, 2017). Each completed questionnaire serves as a case, resulting in a total of 333 sample cases containing five antecedent variables and one outcome variable. Based on Fiss’s (2011) calibration method for the five-point Likert scale, the mean values of each variable are initially coded as crossover points. The calibration standards for these crossover points are set at the 0.5 percentile, with the standard for fully belonging set at the 0.95 percentile and the standard for fully non-belonging set at the 0.05 percentile. When the raw data matches the calibration anchor points, you can adjust 0.5 to 0.501 or 0.499 to prevent the loss of individual cases. The fsQCA 4.1 software’s calibrate function is then used to transform the variable values into fuzzy scores ranging from zero to one, resulting in the calibrated data.

### 4.2. Necessary Conditions Analysis

Necessary condition testing was conducted using fsQCA 4.1 software. The analysis of necessity for single-factor variables determines whether an individual factor is a necessary condition for the outcome’s occurrence and forms the basis for a fuzzy-set qualitative comparative analysis. Due to the asymmetry of causality, a necessary condition analysis was conducted separately for a high and a low level of learning engagement. Table 1 shows that among the antecedent variables affecting a high level of learning engagement, the consistency levels of professional emotions and professional evaluations exceed 0.9, making them approximate necessary conditions for achieving a high level of learning engagement. In contrast, for antecedent variables affecting a low level of learning engagement, all variables have consistency levels below 0.9, indicating that the students’ learning engagement levels are not determined by a single fixed variable but rather result from complex and concurrent factors. This paper further conducts a sufficiency analysis on these antecedent conditions to identify the complex configurational pathways influencing a high and a low level of learning engagement.

### 4.3. Configurational Path Analysis

Configurational analysis requires selecting thresholds for variable consistency and case frequency to filter out explanatory variables that sufficiently explain the outcome variable. The set frequency threshold should retain at least 75% of the observed cases. When the sample size is large, a higher frequency threshold should be chosen. The case frequency threshold is set at eight, the raw consistency threshold at 0.8, and the PRI (Proportional reduction in inconsistency) consistency threshold at 0.65 (Du & Jia, 2017). This paper primarily focuses on analyzing intermediate solutions and combines simple solutions to determine whether variables are core or peripheral conditions (Greckhamer et al., 2018). To clearly and intuitively present each configurational path, this study follows the typical representation method proposed by Ragin (2008), in which “●” indicates the presence of a core condition, “•” indicates the presence of a peripheral condition, “⊗” indicates the absence of a core condition, and “⊗” indicates the absence of a peripheral condition.

Table 2 shows that there are two configurational paths influencing a high level of learning engagement (H1 to H2) and three configurational paths influencing a low level of learning engagement (NH1a to NH1c). The consistency levels of the configurations of both a high and a low level of learning engagement exceed 0.9, surpassing the acceptable level of 0.8. The overall solution coverage is 0.8354, indicating that the configurational paths have a strong explanatory power for learning engagement outcomes.

#### 4.3.1. Configurational Paths for a High Level of Learning Engagement 

Based on the characteristics of the covered cases, the configurational paths for a high level of learning engagement are divided into two categories: the endogenous model and the endogenous–exogenous promotion model. Configurational path H1 indicates that positive professional evaluations and high academic self-efficacy are the core conditions, while high levels of professional cognition and professional emotions are peripheral conditions. Regardless of whether students perceive a supportive atmosphere from the university environment, as long as they possess strong knowledge of and efficacy in their tourism major, they can achieve a high level of learning engagement. In this case, internal factors play a decisive role.

Configurational path H2 indicates that positive professional evaluations and a high level of university support are the core conditions, while high levels of professional cognition and professional emotions are peripheral conditions. For students in this category, regardless of their academic self-efficacy, as long as they have a strong identification with the tourism major and perceive the university’s support for their learning and a positive atmosphere at the university, they can achieve a high level of learning engagement. This reflects the combined influence of both internal and external factors; however, external environmental factors are also the result of students’ subjective perceptions.

#### 4.3.2. Configurational Paths for a Non-High Level of Learning Engagement

Based on the core deficiency conditions, the configuration paths for a low level of learning engagement are divided into three but can be grouped into one major category, termed the “Internal constraint model”. First, non-high professional emotional engagement is a core non-identification condition for all three paths, indicating that if tourism students lack an emotional connection to their major, even if other conditions are present or multiple conditions are met, a high level of learning engagement cannot be achieved. Second, a low level of academic self-efficacy is a marginal deficiency condition, indicating that students who lack confidence in their ability to use their skills to complete their tourism studies will suppress their learning states and performance. Third, aside from the aforementioned missing conditions of professional emotions and academic self-efficacy, the three paths each represent different types of students. On the basis of the suppression of both professional emotions and academic self-efficacy, other internal and external factors further suppress the learning engagement of different types of students, primarily due to the suppression of internal psychological factors.

### 4.4. Robustness Testing

In accordance with relevant research methods (Du et al., 2020), two methods were used for robustness testing. First, the consistency threshold was adjusted by changing the PRI consistency threshold from 0.80 to 0.85; second, some cases were deleted, and the remaining cases were analyzed. The configuration results of the two testing methods did not show significant changes and were generally consistent with the original configurational results, indicating that the findings of this study are robust.

### 4.5. Analysis of Configurational Influence

#### 4.5.1. Analysis of Configurational Influence on a High Level of Learning Engagement

Having positive professional evaluations is the core condition for a high level of learning engagement, while high levels of professional cognition and professional emotions are peripheral conditions. In the configurational paths for a high level of learning engagement, having a strong professional identity is a common core condition in two paths, indicating that professional identity has a significant impact on the learning engagement of tourism major students. Professional identity has always been a key aspect of higher education development and is a crucial indicator for measuring learners’ personal values and career development goals (Bai et al., 2012; Stefanini et al., 2021). One’s professional identity is increasingly understood as a dynamic psychological process evolving from professional cognition and professional evaluations to professional emotions. Similar to the spiraling process of “I know”, “I am suitable”, and “I like”, all three dimensions significantly impact the learning engagement of tourism students. However, the three psychological dimensions have varying effects on learning engagement. Table 2 shows that professional evaluations are the core condition, while professional cognition and professional emotions are peripheral conditions. This indicates that “suitability for oneself is the best”, a prerequisite and core condition for achieving a high level of learning engagement. Cognitive appraisal theory (CAT) suggests that cognitive appraisal affects students’ emotional states and that evaluation is an important factor in the development of emotion during the learning process (Dodd et al., 2011; Roseman et al., 1990).

**Figure 2:**
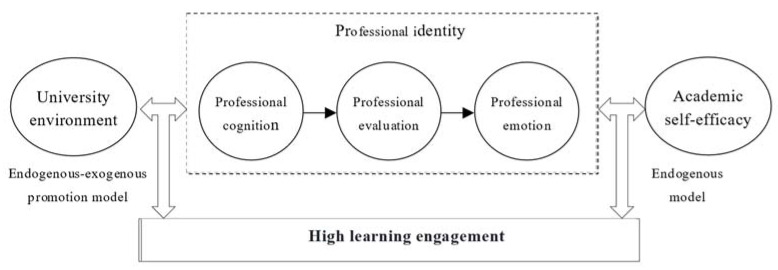
Configurational paths of a high level of learning engagement.

The “endogenous model” and “endogenous-exogenous promotion model” paths are two routes to achieving a high level of learning engagement, with the original and unique coverage of these paths being essentially the same. A comparison of the two configurational paths shows that in the case of H1 (professional identity * academic self-efficacy), tourism students who maintain a strong professional identity also need to possess appropriate levels of positive academic self-efficacy to adopt a positive and proactive attitude towards learning, continuously enhance their academic self-efficacy, and maintain sustained learning motivation, which in turn affects learning outcomes. In the case of H2 (professional identity * university environment), tourism students with a strong professional identity also benefit from their perception of the learning environment and atmosphere provided by the university. This includes the course content, job opportunities, educational facilities, campus atmosphere, and teacher–student relationships. These factors effectively guide students in their learning, enhance their sense of belonging to both the profession and the university, and contribute to a high level of learning engagement, as illustrated in Figure 2.

#### 4.5.2. Analysis of Configurations for a Low Level of Learning Engagement 

Table 2 shows that although there are three configurational pathways for a low level of learning engagement, they can be grouped into a single type. This is because all three paths indicate that having a low level of professional emotions is a core non-identification condition for a low level of learning engagement, while having low academic self-efficacy is a marginal deficiency. The analysis of the three dimensions of professional identity mentioned above shows that the results of students’ evaluations of their major may lead to either emotional acceptance and attachment to the tourism major or difficulty in developing such emotions, which in turn could suppress learning engagement. Table 2 demonstrates that the lack of emotional attachment to the major among tourism students serves as a “core non-identification condition” of a low level of learning engagement. According to configurational theory, this finding suggests that improving learning engagement would be challenging, even when other internal and external factors are employed, if emotional attachment to the major is absent. The significant role of emotional attachment to the major in shaping undergraduates’ learning engagement has been further substantiated by prior research (Q. Chen et al., 2024; Yu et al., 2021). This process aligns with the principles of the “cognitive-emotion-behavior” theory, which posits that cognitive processes influence emotional responses, and these emotional changes, in turn, impact behavior, including students’ learning engagement (Roseman et al., 1990; Parker et al., 2021). The above indicates that professional emotions are a key factor in having a low level of learning engagement. To address the learning issues faced by tourism students, rather than focusing on enhancing their positive and well-adjusted motivation and engagement levels, it may be more effective to consider how to reduce their negative and maladaptive motivation and engagement behaviors. This involves implementing an “education of love” for the tourism major to encourage “poorly adapted learners” to develop ideal emotional transformations.

A low academic self-efficacy further suppresses the degree of learning engagement. Overall, academic self-efficacy reflects an individual’s self-assessment of their interaction with the environment. Tourism students with strong academic self-efficacy are interested in new challenges and fully engage in them, continuously striving to overcome difficulties in their learning process. During this process, their academic self-efficacy will also be continually reinforced and enhanced. If students lack an emotional connection to the tourism major, are unable to regulate their internal and external environments, and lack the confidence or beliefs necessary to learn, such as the belief “I can do it”, their enthusiasm for engaging in learning will continually diminish. Additionally, Table 2 shows that having low levels of professional cognition, negative professional evaluations, and a poor university environment all exert certain inhibiting effects on the three pathways of low levels of learning engagement.

## 5. Discussion and Conclusions

### 5.1. Conclusions

This study was conducted against the backdrop of the demand for high-quality development of tourism education in China. The conclusions are as follows:

(1) Learning engagement results from the combined effects of internal and external factors, with internal factors (professional cognition, professional evaluations, professional emotions, and academic self-efficacy) playing a dominant role and external factors (university environment) providing supportive effects. However, the impact of these factors varies among different types of students, and external factors often exert their influence through internal factors. The level of academic engagement is the result of the combined influence of both internal and external factors. External factors, such as the university environment, stimulate the desire to learn, provide direction, and motivate students. Internal factors, such as professional identity, continuously drive learners to engage in deeper learning and overcome challenges. When external and internal factors align, they more effectively stimulate learning efficacy and improve learning outcomes.
(2) There are two pathways for the configurations of a high level of learning engagement: the “endogenous model” (professional evaluations * academic self-efficacy) and the “endogenous-exogenous promotion model” (professional evaluations * university support). Positive professional evaluations are a core condition, while high academic self-efficacy and a high level of university support are marginal conditions, respectively. Professional identity, constructed from the dimensions of professional cognition, professional evaluations, and professional emotions, plays a crucial role in achieving a high level of learning engagement.
(3) The pathways for the configurations of a low level of learning engagement can be categorized into one type with three variations, named the “endogenous suppression model”. Having a low level of professional emotions is the core non-identification condition for a low level of learning engagement, while having low academic self-efficacy is a marginal deficiency condition. The other three factors also exert a certain degree of suppression on different types of learning engagement.

### 5.2. Theoretical Implications

First, this study constructs a configurational impact model of learning engagement for tourism undergraduates based on internal and external factors. Existing research has primarily focused on exploring the factors affecting university students’ learning engagement from either an internal psychological or external environmental perspective (W. Wang & Wang, 2021; Yu et al., 2021). This study points out that learning engagement is a holistic concept that includes both internal psychology and external behavior, influenced by both internal and external factors (H. Yin, 2020). By organizing and summarizing internal factors such as professional identity (professional cognition, professional evaluations, and professional emotions) and academic self-efficacy, along with external factors like the university environment, this study constructs a configurational impact model consisting of five factors. The empirical research results show that the learning engagement of tourism undergraduates is driven by both internal and external factors, validating the applicability and rationality of the model. This enriches the application of the learning engagement theory and configurational theory in tourism education research, potentially moving toward Kahu’s (2013) ideal classification system that merges various viewpoints into a unified research perspective on students’ learning engagement.

Second, identifying the configurational impact models of high (or low) levels of learning engagement reveals the core factors influencing learning engagement among tourism students. The research findings reveal an interesting and valuable conclusion: there are differences in the core influencing factors between a high level of learning engagement and a low level of learning engagement. Existing studies often overlook comparisons and distinctions between the two (Q. Chen et al., 2024; Yu et al., 2021). Table 2 shows positive professional evaluations serve as a fundamental and predictive factor for a high level of learning engagement, while having a low level of professional emotions functions as a fundamental and predictive factor for a low level of learning engagement. This validates an interesting proverb: “What suits you best is the best” and “All giving up stems from a lack of passion”. It demonstrates that there are differences in the impact paths between a high level of learning engagement and a low level of learning engagement, which has important implications for policy formulation. Policies designed for a high level of learning engagement may not necessarily improve a low level of learning engagement; therefore, strategies should be tailored separately for the two types of students.

Third, understanding the differences and interactions between internal and external factors affecting learning engagement is crucial. This study shows that internal factors are the primary reasons affecting learning engagement, while external factors like the university environment are secondary. Furthermore, external factors also need to exert their effects through a learner’s self-perception. Professional identity is a progressive dynamic process consisting of professional cognition, professional evaluations, and professional emotions. The extent of impact of these three factors on high (or low) levels of learning engagement varies, but their combined effect interacts to influence learning engagement. This highlights the significant importance of professional identity education in addressing issues in tourism education (J. Wang, 2018). Academic self-efficacy is one of the core conditions for a high level of learning engagement and a marginally missing condition for a low level of learning engagement. This indicates that cultivating academic self-efficacy has a significant impact on the learning of tourism students (Guo et al., 2021), especially as digital transformations drive innovation in higher education, which may result in a scenario in which “the strong get stronger” and “the weak get weaker” in terms of student academic self-efficacy. The university environment is also one of the core conditions for a high level of learning engagement (Tian, 2018). However, the support or suppression of learning engagement by the external environment is a result of the students’ self-perception. The effect of the external environment on learning engagement may vary from person to person. A good environment does not necessarily lead to positive perceptions, and a poor environment might even motivate students to overcome difficulties and enhance their learning drive.

### 5.3. Practical Implications

First, establish a holistic view of learning engagement, focusing on the interplay between internal and external factors to collectively drive learning engagement. Zepke (2015) suggests that college students’ learning engagement should be viewed as a socio-cultural ecosystem connecting the classroom, students’ personal backgrounds, and society at large. Similarly, Kahn (2014) emphasizes the impact of individual intrinsic factors and external social relationships on students’ learning engagement. Therefore, understanding the learning engagement of tourism students requires a holistic perspective that spans both internal and external factors. This study also shows that intrinsic psychological factors and external environmental factors jointly influence the level and performance of learning engagement. We should also develop policies to promote student engagement from both internal and external aspects.

Second, implement targeted measures to improve students’ alignment with the tourism major, cultivate professional emotions, and enhance professional identity. This study shows that significant differences exist in the factors influencing a high level of learning engagement and a low level of engagement. It is essential to adopt different educational methods and strategies based on individual student differences and their levels of learning engagement to promote each student’s development. On the one hand, focus on students’ personalities, needs, and the goals of the tourism program. Analyze the alignment between individual students and the tourism major, and guide students to adjust and enhance their fit with the major by comparing the costs and potential benefits. On the other hand, utilize scientific methods such as personality tests, psychological counseling, and career guidance to understand and care for students. Help them gain thorough self-awareness, recognize their strengths and advantages, and understand how to leverage these strengths within their major. This will strengthen their understanding of the profession, enhance their professional fit, cultivate students’ emotional connections, and improve their sense of professional identity (Cooper et al., 2017).

Third, boost students’ self-confidence, enhance their ability to face difficulties and solve problems, and foster academic self-efficacy. Academic self-efficacy determines how university students respond to psychological challenges such as learning difficulties and anxiety. Students with higher levels of academic self-efficacy can effectively engage in psychological regulation and adapt to changes in internal and external environmental factors. This positively impacts their problem-solving flexibility and initiative, which in turn affects their learning activities and outcomes. However, research indicates that Chinese university students tend to have lower levels of academic self-efficacy, which affects their learning engagement and results, leading to lower self-evaluations of their academic performance and abilities (Bao, 2023). Therefore, universities need to strengthen the cultivation of interdisciplinary thinking skills to empower the development of students’ academic self-efficacy (K. D. Su, 2024). They should encourage positive interactions among peers, faculty members, and technology, providing encouragement and guidance for students. This will help students discover their strengths and talents, build their confidence and belief in themselves to overcome difficulties and solve problems, and continuously enhance the academic self-efficacy of students in tourism programs.

Fourth, strengthen university support to enhance students’ sense of belonging and guide their learning behaviors. The university environment helps cultivate students’ cultural resilience and academic interests, thereby increasing their pride in and attachment to the university. Therefore, universities should strive to create a favorable learning environment both inside and outside the classroom. Firstly, outside the classroom, universities should allocate educational resources effectively, increasing levels of academic support for students in areas such as credit systems, undergraduate mentorship, domestic and international exchanges, social practices, extracurricular activities, and professional competitions. Secondly, inside the classroom, teachers should adhere to a student-centered teaching approach (Kim & Davies, 2014), actively communicate with students, promptly identify difficulties they encounter in life and learning, and provide timely assistance. In summary, by offering a variety of learning, practice, and academic exchange activities, universities can create an excellent learning environment and foster a harmonious and supportive campus culture, which will bridge the psychological distance between teachers and students, as well as among students themselves, enhancing their sense of belonging and fulfillment. This, in turn, will influence students’ learning motivation and behaviors, ultimately improving their individual learning outcomes and the quality of their learning.

### 5.4. Research Limitations and Future Research

This study has several limitations that should be addressed in future research. First, while this research explores the configurational impact paths of learning engagement, it does not specifically analyze the psychological and behavioral characteristics of students across different paths. Future studies should further analyze and compare the characteristics of students in these different paths and conduct a more in-depth exploration of the five influencing factors to gain a more comprehensive understanding of the multidimensional aspects of learning engagement.

Second, there are significant regional and developmental differences in both the tourism industry and tourism higher education in China. Therefore, future research could focus on tourism students from different regions, university types, and academic backgrounds to conduct more comprehensive and in-depth investigations. This would allow for a comparative analysis of the differences in learning engagement characteristics and their influencing factors, further validating the results of this study, especially in terms of regional variations in student engagement.

Third, while this study constructs a configurational influence framework of learning engagement that includes both internal and external factors, it suggests that the socio-cultural environment in China may significantly impact the learning engagement of tourism students. Future research could integrate socio-cultural variables into the model to provide a more comprehensive interpretation of the relationship between learning engagement and socio-cultural factors. Specifically, future studies could explore how factors such as collectivism, educational values, and social identity within the Chinese cultural context affect tourism students’ learning engagement. This could provide a more nuanced understanding of how external socio-cultural factors interact with internal academic and professional factors in shaping students’ engagement.

Finally, snowball sampling was employed to recruit participants, which, while effective for reaching the target population, may have introduced sampling bias. The non-random nature of snowball sampling means that the sample may not fully represent the broader population of tourism students, as it may have overrepresented certain social or academic groups within the student body. Future studies could consider using random sampling techniques to improve the representativeness of the sample and enhance the generalizability of the findings.

## Figures and Tables

**Table 1:** Results of necessary conditions analysis.

Conditional Variables	A High Level of Learning Engagement		A Low Level of Learning Engagement	
	Consistency	Coverage	Consistency	Coverage
Professional cognition	0.877005	0.810437	0.580616	0.552970
~ Professional cognition	0.516251	0.544297	0.800958	0.870322
Professional evaluation	0.910544	0.812538	0.593219	0.545574
~ Professional evaluation	0.490762	0.539302	0.796165	0.901696
Professional emotion	0.918410	0.804455	0.589077	0.531781
~ Professional emotion	0.465456	0.523598	0.783385	0.908218
Academic self-efficacy	0.880603	0.844453	0.573043	0.566342
~ Academic self-efficacy	0.547778	0.554540	0.842612	0.879128
University environment	0.890909	0.824260	0.590497	0.563047
~ University environment	0.527716	0.555634	0.815690	0.885136

**Table 2:** Configuration paths of learning engagement.

Conditions	A High Level of Learning Engagement		A Low Level of Learning Engagement		
	H1	H2	NH1a	NH1b	NH1c
Professional cognition	•	•	—	⊗	⊗
Professional evaluation	●	●	⊗	—	⊗
Professional emotion	•	•	⊗	⊗	⊗
Academic self-efficacy	●	—	⊗	⊗	⊗
University environment	—	●	⊗	⊗	—
Consistency	0.927805	0.923514	0.978367	0.973772	0.978367
Raw coverage	0.770352	0.771633	0.652919	0.64144	0.644517
Unique coverage	0.0358558	0.0371366	0.0358558	0.0371366	0.0272766
Overall consistency	0.912046		0.961554		
Overall coverage	0.807489		0.704395		

## Data Availability

The data presented in this study are available on request from the corresponding author due to privacy restrictions.

## Appendix A

**Table A1:** Measurement scales.

Construct	Measurement Items
**Professional cognition**	I know what the tourism program requires of students.
	I understand the employment situation in the tourism program.
	I know what the outside world says about the tourism program.
	I know the position of tourism program in our university.
	In general, I have a clear understanding of the tourism program.
**Professional evaluations**	I have good professional thinking in tourism.
	My personality matches my program in tourism.
	The tourism program can reflect my expertise.
	I am persistent in my study of tourism.
	I am well suited to study tourism.
	I feel at ease studying tourism.
**Professional emotions**	I am willing to engage in work related to the tourism program.
	I have accepted the tourism program in my heart.
	I haven’t thought about changing my program.
	I have a positive evaluation of the tourism program.
	I have great confidence in the development prospects of the tourism program.
	I have developed positive feelings towards the tourism program.
	I am satisfied with the overall situation of the tourism program at our university.
	In general, I like tourism programs.
**Academic Self-Efficacy**	I can always manage to solve difficult problems in my studies if I try hard enough.
	If someone opposes me, I can find means and ways to get what I want in studying.
	It is easy for me to stick to my study aims and accomplish my academic goals.
	I am confident that I could deal efficiently with unexpected events during my studies.
	Thanks to my resourcefulness, I know how to handle unforeseen situations in study.
	I can solve most study problems if I invest the necessary effort.
	I can remain calm when facing difficulties in studying because I can rely on my coping abilities.
	When I am confronted with a problem in my study, I can usually find several solutions.
	If I am in a bind, I usually have something to do.
	No matter what comes my way, I’m usually able to handle it.
**University Environment**	My university offers professional courses on tourism.
	My university offers project work focused on tourism.
	My university offers internship focused on tourism.
	My university offers a bachelorʹs or masterʹs degree in tourism.
	My university arranges conferences/workshops on tourism.
	My university brings tourism students into contact with each other.
	My university creates awareness of tourism as a possible career choice.
	I feel very comfortable with the overall teaching and learning environment, atmosphere, equipment, and facilities of my university.
	The library of my university has abundant and authoritative information and an excellent environment, which can meet my learning needs.
	I can always get notice and information related to my tourism program in a timely and convenient way.
	The teachers/mentors at my university will take the initiative to praise and affirm my progress in my tourism studies.
	The teachers/mentors at my university will actively listen to my confusion in tourism studies and give me comfort.
	The faculty or teaching support staff of my university will give me advice and guidance for further study.
**Learning Engagement**	I am very resilient mentally, as far as my studies are concerned.
	I feel strong and vigorous when I’m studying or going to class.
	When I’m doing my work as a student, I feel bursting with energy.
	I am enthusiastic about my studies.
	I am proud of my studies.
	I find my studies full of meaning and purpose.
	When I am studying, I forget everything else around me.
	I am immersed in my studies.
	It is difficult to detach myself from my studies.
